# Integrated computational analysis of molecular mechanisms underlying perfluorooctane sulfonic acid induced thyroid toxicity

**DOI:** 10.1038/s41598-025-92678-2

**Published:** 2025-03-06

**Authors:** Haoran Li, Bo Yu, Ye Yuan, Nannan Chen, Huicai Guo, Haiqiang Zhang, Zhiqing Zhang

**Affiliations:** 1 Department of Pharmacy, The Second Hospital of Hebei Medical University, 215 Heping West Road, Shijiazhuang, 050000 Hebei Province China; 2 Department of Pediatrics, The Second Hospital of Hebei Medical University, Shijiazhuang, 050000 China; 3 School of Pharmacy, Hebei Medical University, Shijiazhuang, 050017 China; 4 Department of Toxicology, School of Public Health, Hebei Medical University, Shijiazhuang, 050017 China; 5 Department of Gastroenterology, The Second Hospital of Hebei Medical University, 215 Heping West Road, Shijiazhuang, 050000 Hebei Province China

**Keywords:** Perfluorooctane sulfonic acid, Thyroid toxicity, Network toxicology, Molecular Docking, Molecular dynamics simulation, Computational biology and bioinformatics, Environmental sciences, Endocrinology

## Abstract

Perfluorooctane sulfonic acid (PFOS), a persistent organic pollutant, significantly disrupts thyroid function. This study presented an integrated computational approach, combining network toxicology, molecular docking, and molecular dynamics simulations to systematically elucidate the molecular mechanisms underlying PFOS induced thyroid toxicity. Through integrated analysis of the Comparative Toxicogenomics Database (CTD), GeneCards, and Online Mendelian Inheritance in Man (OMIM) databases, we identified 205 potential thyroid toxicity-related targets. Protein-protein interaction network analysis revealed 34 hub targets, with TP53, JUN, ESR1, AKT1, and CTNNB1 emerging as central nodes in the toxicity network. Functional enrichment analysis demonstrated significant enrichment in the PPAR signaling pathway, fatty acid metabolism, AGE-RAGE pathway, and AMPK pathway, indicating that PFOS influences thyroid function through multiple signaling pathways. Molecular docking studies showed that PFOS forms stable complexes with core target proteins, with binding energies ranging from − 4.9 to -9.7 kcal/mol. Molecular dynamics simulations further validated the structural stability of these complexes, with PFOS-AKT1 and PFOS-TP53 exhibiting the highest conformational stability. This study revealed the multi-target and multi-pathway characteristics of PFOS-induced thyroid toxicity, providing novel insights into its toxicological mechanisms.

## Introduction

Perfluorooctane sulfonic acid (PFOS), a representative perfluoroalkyl substance (PFAS), has been extensively utilized in industrial applications due to its excellent surface activity and chemical stability^1,2^. However, the persistence, bioaccumulation, and long-range migration characteristics of PFOS have elevated it to an environmental contaminant of global concern^3^. Environmental monitoring data in recent years show that PFOS is ubiquitous in various environmental media worldwide, including surface water, sediments, atmosphere, and organisms^4–6^. Notably, PFOS has been detected in over 95% of human populations, with surveys in the United States reporting geometric mean serum concentrations of 7–10 ng/mL in adolescents and adults^7^. In contrast, European Union populations exhibit mean serum levels of 7.7 ng/mL in adults and 3.2 ng/mL in children^8^.

Studies have established that PFOS demonstrates a mean serum half-life ranging from 3.4 to 5.7 years^9^, and its persistence and bioaccumulate nature pose substantial threats to human health^10^. Extensive research has demonstrated a strong correlation between PFOS exposure and various adverse health outcomes. Studies have shown that PFOS exposure is significantly associated with health problems, including abnormal liver function, elevated cholesterol levels, reduced fertility, cancer, and impaired neurodevelopment in children^11^. In addition, PFOS has been shown to have endocrine disrupting effects, which can interfere with the normal secretion and metabolism of multiple hormones^12^.

The thyroid gland, a crucial endocrine organ, plays a pivotal role in regulating metabolism, growth and development, and neurological functions^13^. Studies have indicated that the thyroid exhibits heightened sensitivity to environmental pollutants, rendering it particularly vulnerable to functional disruption^14^. Epidemiological investigations have revealed significant correlations between PFOS exposure levels and alterations in thyroid hormone concentrations^15,16^. Animal studies have further substantiated these findings, suggesting that PFOS exposure can perturb multiple aspects of thyroid hormone homeostasis, including synthesis, transport, and metabolism^17,18^.

In recent years, extensive research efforts have been made to elucidate the mechanisms underlying PFOS’s effects on the thyroid system. In vivo studies have demonstrated that PFOS exposure significantly modulates serum thyroid hormone levels, including alterations in thyroid stimulating hormone (TSH), thyroxine (T4), and triiodothyronine (T3) concentrations^19,20^. Experimental investigations have revealed multiple pathways through which PFOS interferes with thyroid function, including inhibition of the crucial thyroid hormone synthesis enzyme, disruption of thyroid hormone transport protein binding, and perturbation of thyroid hormone receptor-mediated signaling pathways^18,21,22^. Additionally, research has implicated oxidative stress and mitochondrial dysfunction as potential mechanisms of PFOS induced thyroid cell damage^23^. However, the current understanding of PFOS induced thyroid toxicity mechanisms remains constrained by several limitations. Primarily, existing studies have focused on isolated targets or signaling pathways, lacking comprehensive toxicity network analysis^24^. Furthermore, the interaction patterns between PFOS and key protein targets remain incompletely characterized^17,25^. Moreover, current methodologies frequently prove inadequate for capturing PFOS induced dynamic molecular events, constraining deeper mechanistic insights^26^.

Computational toxicology methods have emerged as increasingly vital tools in elucidating the toxicity mechanisms of environmental pollutants. Network toxicology, by integrating multi-omics data and systems biology approaches, enables comprehensive revelation of molecular networks underlying toxicant actions^27^. Studies have shown that transcriptomic analysis can identify differentially expressed gene networks induced by PFOS exposure, facilitating the prediction of critical toxicity pathways^28^. In addition, protein-protein interaction network analysis enables the identification of upstream regulatory factors and downstream effector molecules in toxicity cascades^29^. Lin et al. employed network toxicology and molecular docking technology to explore the potential carcinogenic toxicity and mechanisms of PFAS in thyroid, kidney, and testicular cancers, revealing the effects of PFAS on relevant genes and proteins^30^. This systematic analytical approach provides a novel perspective for understanding complex toxicological responses.

Molecular docking, a powerful tool for investigating compound-protein interactions, has found significant applications in toxicity target identification. Developing high-quality protein structure models and optimized docking algorithms facilitates the precise prediction of ligand binding sites and conformations^31^. Previous studies have successfully predicted the binding mode of PFOS with thyroid hormone transport protein using molecular docking, elucidating its competitive binding mechanism^32^. Lu et al. discovered that placental transfer of PFAS correlates strongly with their interactions with human serum albumin and organic anion transporter 4^33^. Additionally, structure-activity relationship studies based on molecular docking have contributed to understanding the relationship between the structural characteristics of PFOS and its toxic effects^34^.

Molecular dynamics simulations provide atomic-level detailed information for understanding the dynamic processes of ligand-receptor interactions. Peng et al. conducted a comprehensive investigation into the binding mechanism and affinity between PFOS and human serum albumin (HSA) through an integrated approach combining multi-spectroscopy, density functional theory, and molecular dynamics simulations. The findings revealed that the binding of HSA-PFAS primarily occurs through hydrogen bonding and hydrophobic interactions, with specific binding site characteristics^35^. This approach provided crucial insights into the transport and distribution of PFOS in vivo. Almeida et al. employed molecular dynamics simulations to explore the interactions between PFAS and PPARγ/RXRα-DNA complexes, revealing the molecular mechanism by which PFAS alters transcriptional activity by affecting nuclear receptor conformational dynamics^36^. Tiburtini et al. utilized computational approaches to predict the interactions of PFAS with human transport proteins, offering novel perspectives on the accumulation of PFAS in humans^37^.

The integrated application of these computational methodologies has substantially advanced our understanding of complex toxicity mechanisms. By constructing potential pathways and target networks through network toxicology, combined with dynamic analyses from molecular docking and molecular dynamics simulations, we can elucidate toxicity mechanisms from systemic to molecular levels. Although these computational predictions provide valuable guidance for subsequent experimental validation, systematic investigations into PFOS-induced thyroid toxicity require further development, particularly regarding the integrated analysis of multi-target and multi-pathway interactions^30,38^.

Therefore, this study aims to elucidate the mechanisms of PFOS-induced thyroid system toxicity through the integration of network toxicology, molecular docking, and molecular dynamics simulations. This investigation will provide novel scientific insights into the potential interference pathways and targets of PFOS at the molecular level. This will contribute to improving the systematic assessment of its health risks and informing future environmental management and public health policies.

## Methods

### Target screening

#### Screening for PFOS-related targets

The Comparative Toxicogenomics Database (CTD) was employed as the data source for identifying PFOS-related targets. CTD represents one of the most comprehensive databases integrating chemical-gene-disease associations, incorporating extensive experimental data on relationships between environmental chemicals and biological targets. This database encompasses a wide range of multidimensional information, spanning the fields of toxicology, gene expression, and disease associations. It has been widely utilized for the purposes of predicting toxicity mechanisms and identifying potential targets^39^.

Target screening was initiated using “Perfluorooctane sulfonic acid” as the search term to identify potential PFOS targets within the CTD database. To enhance the biological significance of target selection, we implemented “Interaction count” as a screening criterion, which quantifies the frequency of literature support for PFOS-target gene interactions. The rationale for this threshold criterion is that higher “Interaction count” values indicate more substantial support for PFOS-gene interactions from the literature and experimental data, representing enhanced reliability and biological relevance. We adopted an incremental threshold strategy to achieve an optimal balance between candidate target quality and quantity, considering both the number of potential targets and research feasibility.

#### Screening for thyroid toxicity-related targets

To comprehensively identify genes associated with thyroid toxicity, this study employed multiple authoritative databases in an integrated target screening approach. Initially, we conducted searches in the GeneCards database using the keywords “Thyroid disorders,” “Thyroid disease,” and “Thyroid dysfunction.” To ensure the reliability of the screening targets, genes with “Relevance Scores” exceeding the median value were selected as candidate targets. Concurrently, we queried the Online Mendelian Inheritance in Man (OMIM) database to identify thyroid function-related genes. OMIM database provides rigorously validated gene-disease association information, with a particularly robust reference value for endocrine system disorders^40^. By integrating information from these diverse databases, this study established a comprehensive thyroid toxicity-related gene repository. Venn diagram analysis was employed to identify the intersection between PFOS targets and thyroid toxicity targets. The genes present in both sets were considered potential targets for PFOS-induced thyroid toxicity. These genes have experimental evidence supporting their interaction with PFOS and are also closely related to the regulation of thyroid function.

### Network toxicology analysis

#### Mapping protein interaction networks

Utilizing STRING database resources, this study established networks illustrating the interactions between target proteins. The STRING database integrates protein interaction information from multiple sources, including experimentally validated interactions, co-expression data, genomic context information, and literature mining results^41^. Potential targets identified for PFOS-induced thyroid toxicity were imported into the STRING database for comprehensive analysis. To ensure the reliability of the network analysis, stringent parameter settings were implemented: the species selection was restricted to “Homo sapiens” to ensure the analysis reflected human protein interaction characteristics and the minimum required interaction score was set to “Highest Confidence (0.9)”, thereby effectively filtering potential false-positive interactions. These rigorous parameter settings ensured the high credibility of the constructed protein-protein interaction network, providing a reliable foundation for subsequent topological analysis. The resultant network comprised nodes (representing proteins) and edges (representing protein-protein interactions), with edge weights derived from STRING’s comprehensive scoring system.

#### Calculation of network topological parameters and core target identification

The protein-protein interaction networks generated from the STRING database were visualized and analyzed using Cytoscape (3.10.2)^42^. Topological parameters for each network node were calculated using the CytoNCA plugin (2.1.6)^43^. We filtered genes according to the primary score file calculated by CytoNCA, where each score of Betweenness Centrality (BC), Closeness Centrality (CC), Degree Centrality (DC), Eigenvector Centrality (EC), Local Average Connectivity (LAC), and Network Topology Coefficient (NC) was higher than the median value^44^. A primary subnetwork was constructed using these filtered genes. The same filtering process was conducted again on this primary subnetwork to acquire the final critical subnetwork, identifying the core targets. This systematic double-filtering approach enables a comprehensive evaluation of node importance within the network through multiple topological parameters.

#### Functional characterization and pathway analysis

To understand the molecular basis of PFOS effects on the thyroid, this study used the clusterProfiler package of the R software (version 4.3.0) for systematic functional analysis^45^. Following the conversion of gene identifiers through org.Hs.eg.db, we examined enrichment patterns across the three major Gene Ontology (GO) categories: biological process (BP), molecular function (MF), and cellular component (CC). Analysis parameters were configured as follows: Benjamini-Hochberg method for *p*-value correction (*p* adjust method = BH), significance thresholds of *p* < 0.05 and q < 0.05, and minimum gene set size of 3 (minGSSize = 3). This study investigated signaling pathway enrichment by leveraging the Kyoto Encyclopedia of Genes and Genomes (KEGG) database resources^46^. KEGG pathway analysis revealed critical signaling pathways involving target genes, facilitating comprehension of the systemic PFOS induced thyroid toxicity mechanism^47^. The following criteria were implemented to ensure analysis reliability: Enrichment Factor > 1.5, adjusted *p*-value < 0.05, and a minimum of three genes per pathway.

The GO and KEGG enrichment analysis results were presented using multiple visualization approaches. For GO analysis, bar plots illustrated significantly enriched functional terms and their associated gene counts across ontology categories (BP, CC, MF), while bubble plots integrated information on gene ratios, significance levels (*p*.adjust), and gene counts. KEGG pathway analysis results were visualized through bar plots displaying significantly enriched pathways and their gene counts, complemented by bubble plots presenting multidimensional enrichment results, including gene ratios, significance levels, and gene counts, with color gradients reflecting significance levels.

### Molecular docking

To predict molecular interactions between PFOS and core target proteins, molecular docking analyses were employed to investigate binding modes and affinities. This study accessed the 3D molecular structure of PFOS through PubChem’s chemical database (https://pubchem.ncbi.nlm.nih.gov) using “perfluorooctane sulphonic acid” as the search term. Energy minimization and PDBQT format conversion were performed using ChemBio3D Ultra (version 14.0.0.117) and AutoDock Tools (version 1.5.7). The crystal structures of screened core target proteins were retrieved from the RCSB Protein Data Bank using the following PDB IDs: TP53 (PDB ID: 2G3R), JUN (PDB ID: 1JUN), ESR1 (PDB ID: 2BJ4), AKT1 (PDB ID: 1H10), and CTNNB1 (PDB ID: 2Z6H). PyMOL (version 2.4.0) removed ligand molecules, water molecules, and non-standard residues. The receptor proteins underwent preprocessing using AutoDock Tools software (version 1.5.7), including polar hydrogen addition, Gasteiger charge calculation, and non-polar hydrogen merging. The grid boxes were configured based on active sites or predicted binding pockets, ensuring complete coverage of potential binding regions. Molecular docking simulations were conducted using AutoDock Vina (version 1.2.2). The conformations exhibiting the lowest docking scores were selected for further analysis, focusing on the interaction types of key residues. The three-dimensional and two-dimensional structures of ligand-receptor complexes were generated using PyMOL (version 2.4.0) and Discovery Studio (version v19.1.0.18287).

### Molecular dynamics simulations

Molecular dynamics simulations were performed using GROMACS (version 2023.2). The amber99sb-ildn force field was used to assign AM1-BCC charges to the PFOS molecule using the antechamber module of AmberTools21, and then the ACPYPE tool was used for GAFF force field parameter generation. The most energetically favorable PFOS-protein complex conformations from molecular docking were selected as initial structures. The complexes were solvated in a cubic box using the TIP3P water model with periodic boundary conditions. The system was neutralized with Na + counter-ions and additional NaCl was added to achieve a physiological concentration of 0.15 M. A multi-stage equilibration protocol was implemented. First, energy minimization was performed using the steepest descent method to eliminate unfavorable contacts. Subsequently, the system underwent 200 ps NVT equilibration followed by 100 ps NPT equilibration. Temperature was maintained at 303.15 K using the V-rescale thermostat, while pressure was controlled at 1 bar using the Parrinello-Rahman barostat.

Production simulations were conducted for 100 ns with a 2 fs time step. Both electrostatic and van der Waals interactions employed a cut-off of 1.0 nm, with long-range electrostatic interactions treated using the Particle Mesh Ewald (PME) method. The LINCS algorithm was used to constrain all bonds. System analysis was performed using the following GROMACS modules: gmx rms for RMSD calculations with protein backbone atoms as reference, gmx gyrate for Rg analysis, gmx sasa for SASA calculations using the probe radius of 1.4 Å, and gmx hbond for hydrogen bond analysis with default distance (3.5 Å) and angle (30°) cutoffs.

## Results

### Targets of PFOS induced thyroid toxicity

**Fig. 1:**
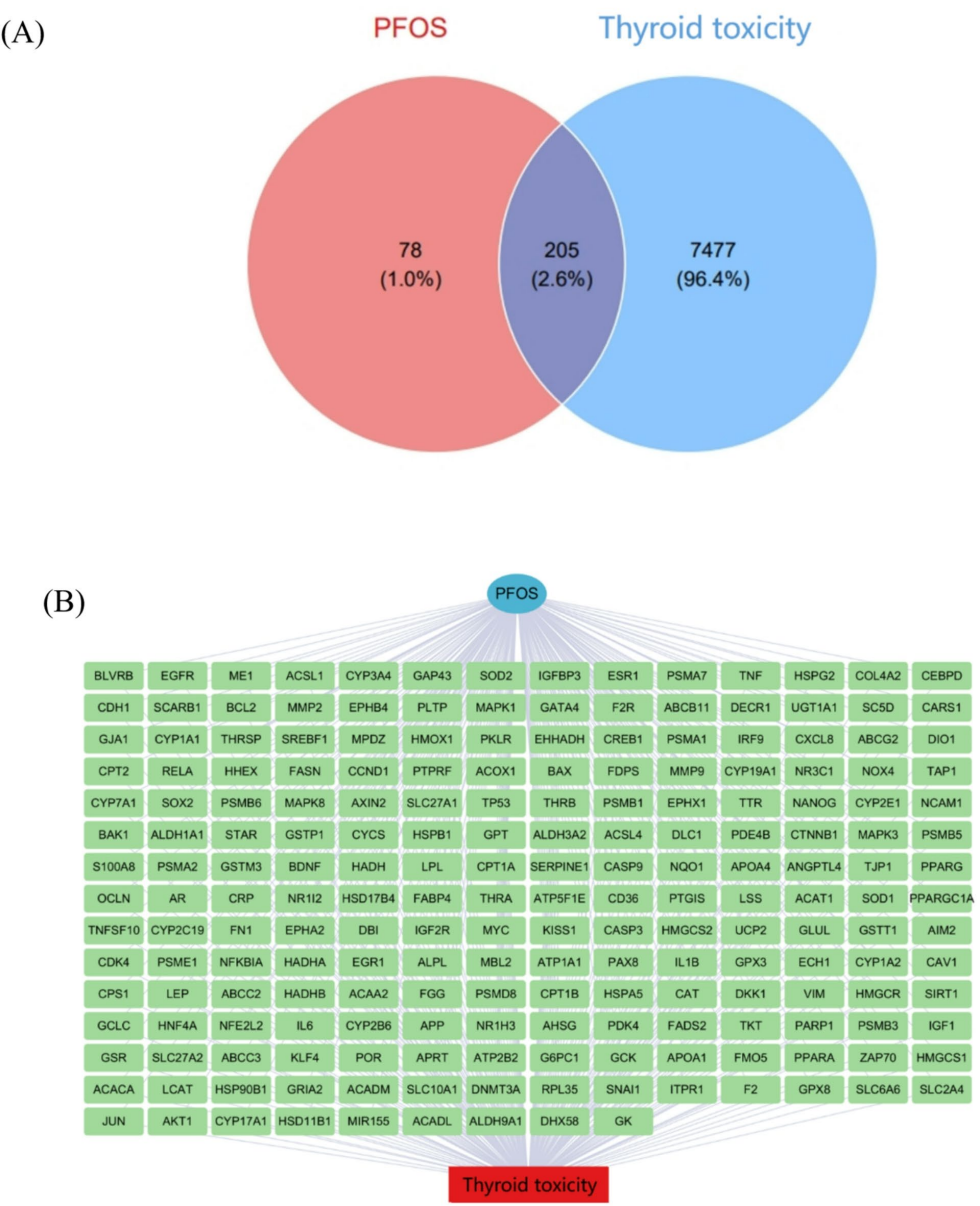
Venn diagram and network relationships of potential targets of PFOS and thyroid toxicity. (**A**) Venn diagram of PFOS and thyroid toxicity targets; (**B**) Network diagram of PFOS-target-thyroid toxicity, green boxes represent genes, blue circles represent PFOS, and red boxes represent thyroid toxicity.

An initial screening of the CTD database for PFOS target genes with an “interaction count” greater than 1 yielded a preliminary target set of 1,841 genes. However, this relatively low threshold potentially incorporated numerous genes with weak PFOS associations, rendering them unsuitable for in-depth analysis. Elevating the threshold to “Interaction count” more significant than three refined the candidate targets to 638, enriching for higher correlation targets. Subsequently, we established a stringent “Interaction count” criterion greater than 4, identifying 283 core targets. This selection criterion ensured high confidence in PFOS-target interactions while balancing biological significance with quantitative manageability. Concurrent analysis of the GeneCards and OMIM databases uncovered 7,682 targets associated with thyroid toxicity. Venn diagram analysis of the intersection between PFOS and thyroid toxicity targets revealed 205 potential thyroid toxicity-related targets (Fig. 1A). The network visualization demonstrates the relationships between these targets, where green boxes represent genes, the blue circle represents PFOS, and red boxes represent thyroid toxicity (Fig. 1B). These targets are substantiated by both experimental evidence of PFOS interaction and established roles in the regulation of thyroid function, suggesting their potential importance in PFOS-induced thyroid toxicity.

### Potential target interaction networks and core gene

**Fig. 2:**
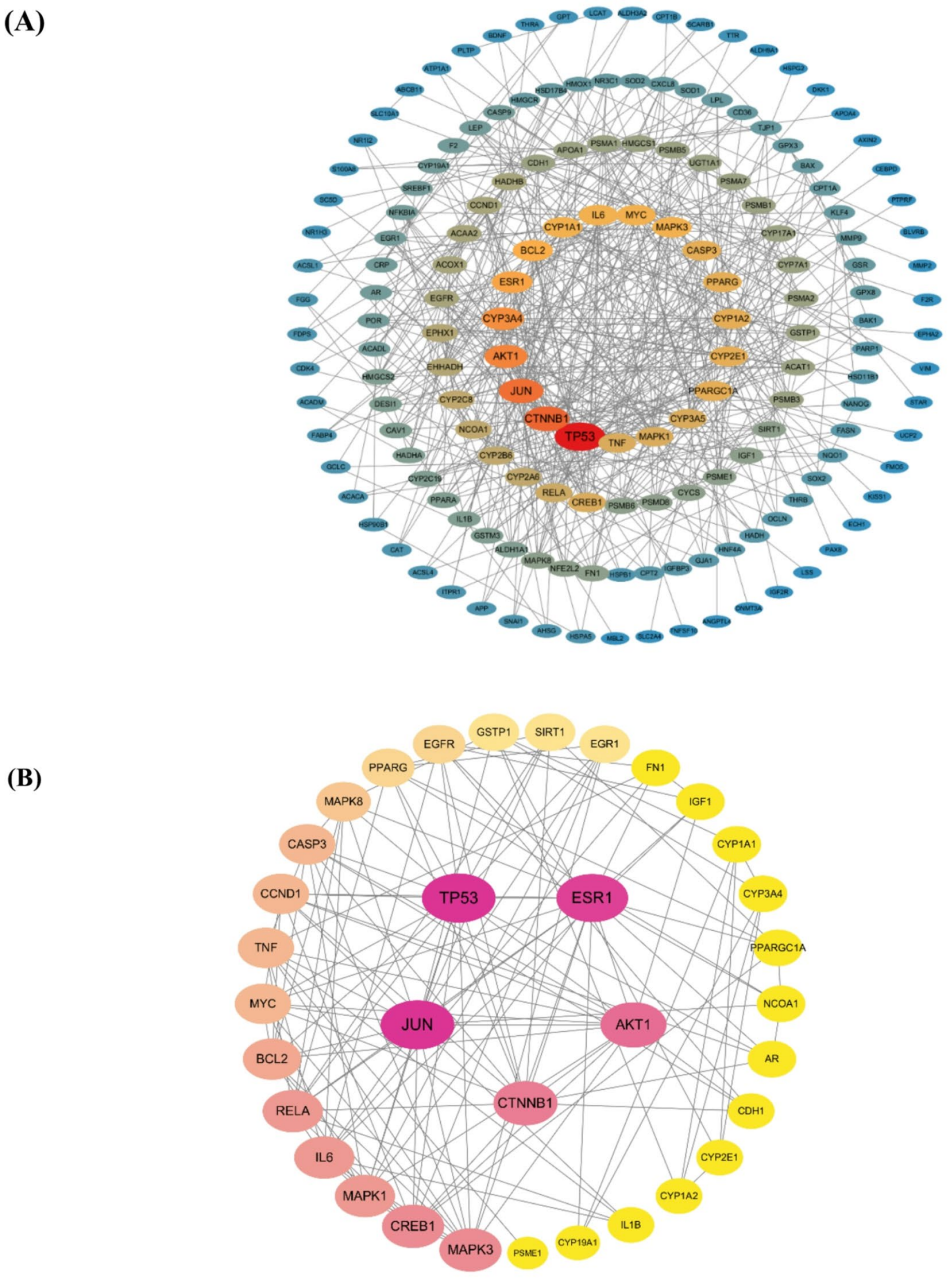
PPI network of potential targets of PFOS induced thyroid toxicity. (**A**) PPI network of protein targets. (**B**) PPI network of core protein targets. The circles represent the target proteins. The larger the diameter of the circle, the brighter the color and the larger the Degree value. The straight lines represent the interactions between the target proteins.

Network analysis via STRING revealed interconnections among 175 protein targets, establishing 535 functional links with an average node connectivity of 8.19. The network exhibited characteristics typical of biological networks, including a network density of 0.035, centralization of 0.231, heterogeneity of 1.124, and a clustering coefficient of 0.405. Network topology analysis was performed to identify core genes critical to the interaction network, focusing on degree and betweenness centrality. An optimized visualization of the PPI network was generated (Fig. 2A), providing a foundation for subsequent target functional analysis and mechanism investigation. Network analysis identified 34 core targets associated with PFOS-induced thyroid toxicity. Network analysis identified 34 core targets associated with PFOS-induced thyroid toxicity. The top five nodes were ranked by degree centrality: TP53, JUN, ESR1, AKT1, and CTNNB1. Network visualization revealed the functional associations among core targets (Fig. 2B). These key regulatory molecules play critical roles in cellular signal transduction and functional regulation, involving biological processes such as cell proliferation, apoptosis, and transcriptional regulation. TP53, a renowned tumor suppressor, orchestrates cell cycle regulation, DNA damage repair, and apoptotic processes. Estrogen receptor α (ESR1), a crucial nuclear receptor, maintains endocrine system homeostasis and potentially mediates PFOS’s endocrine-disrupting effects. JUN, a member of the AP-1 transcription factor family, regulates cell proliferation, differentiation, and apoptosis, with pivotal functions in stress responses. CTNNB1 is a core effector in the Wnt signaling pathway, modulating cell proliferation and differentiation. AKT1, a key kinase in the PI3K/AKT signaling pathway, modulates many biological processes, including cell survival, metabolism, and growth.

### Functional analysis and pathway enrichment of the potential targets

**Fig. 3:**
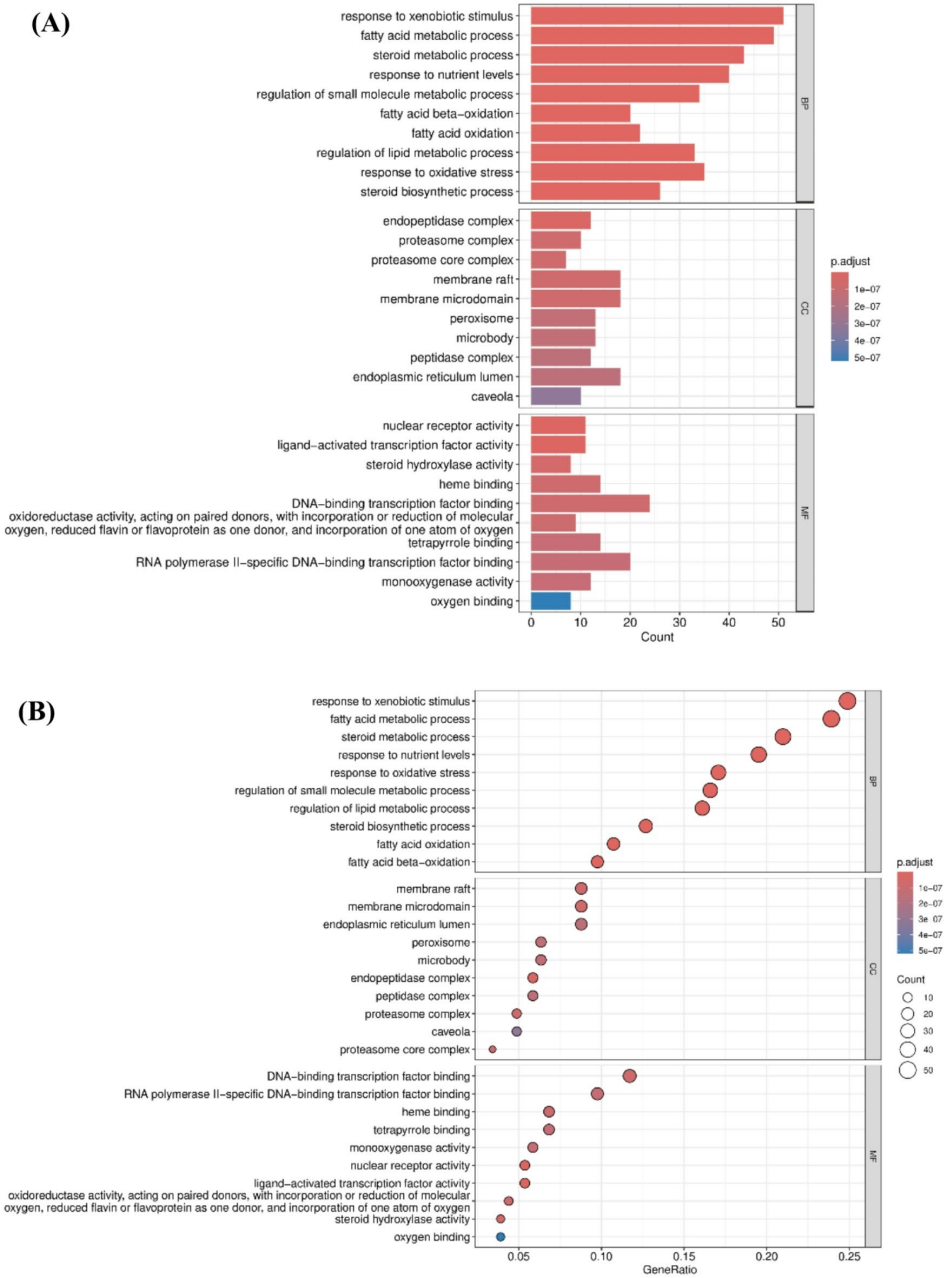
GO enrichment analysis of potential targets of PFOS induced thyroid toxicity. (**A**) GO enrichment analysis bar chart. Different colors represent three GO categories: biological process (BP), cellular component (CC), and molecular function (MF). The x-axis shows the number of genes, and the y-axis shows the significantly enriched GO terms. (**B**) GO enrichment analysis bubble chart. The size of the bubble represents the number of genes, and the color indicates the adjusted *p*-value. The X-axis shows the gene ratio, and the Y-axis shows the significantly enriched GO terms.

GO enrichment analysis was conducted on the overlapping genes between PFOS-associated targets and those linked to thyroid toxicity to investigate the molecular mechanisms of PFOS-induced thyroid effects. The bar chart visualization (Fig. 3A) revealed that in terms of BP, the significantly enriched items involved xenobiotic stimulus response, fatty acid metabolic processes, steroid metabolic processes, and oxidative stress response. The bubble plot (Fig. 3B) further illustrated the significance levels and gene ratios of these enriched terms. Notably, xenobiotic stimulus response showed the highest significance, suggesting that PFOS, as an environmental pollutant, may influence thyroid function through stress response induction. The CC analysis revealed enrichment in endopeptidase complexes, proteasome complexes, membrane rafts, and endoplasmic reticulum lumen, indicating that PFOS may interfere with thyroid function through protein degradation and membrane transport processes. The MF analysis demonstrated enrichment in nuclear receptor activity, ligand-activated transcription factor activity, steroid hydroxylase activity, and oxidoreductase activity. These findings indicate that PFOS may disrupt thyroid hormone synthesis, transport, and metabolism by influencing the activity of transcription factors and critical metabolic enzymes.

**Fig. 4:**
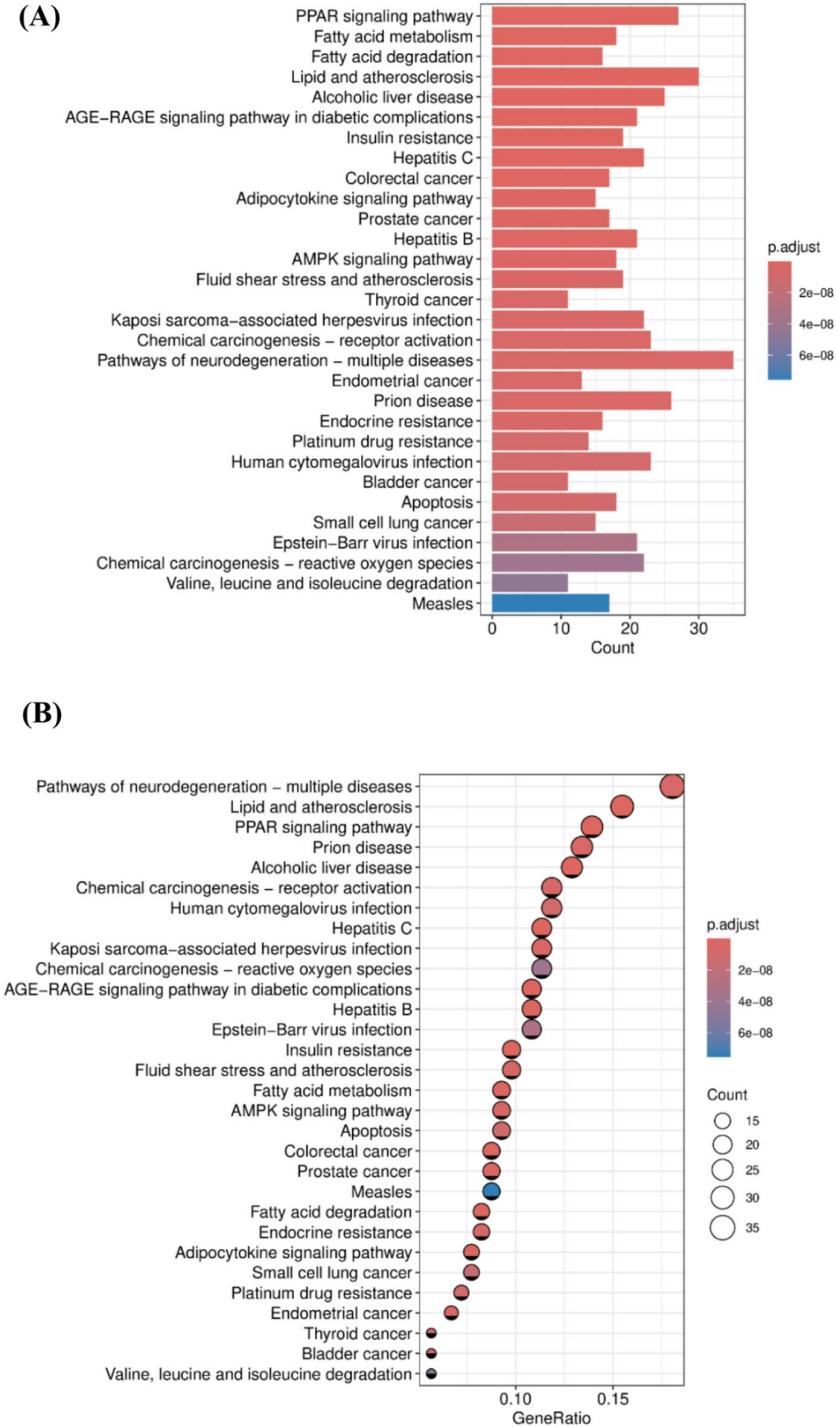
KEGG pathway analysis of potential targets of PFOS induced thyroid toxicity. (**A**) KEGG enrichment analysis bar chart. The X-axis shows the number of genes, and the Y-axis shows the pathway names. Color depth represents the adjusted *p*-value. (**B**) KEGG enrichment analysis bubble chart. The size of the bubble represents the number of genes, and the color indicates the adjusted *p*-value significance. The X-axis shows the gene ratio, and the Y-axis shows the significantly enriched pathways.

KEGG pathway enrichment analysis identified significantly associated signaling pathways, presented as both bar chart (Fig. 4A) and bubble plot visualizations (Fig. 4B). The bar chart revealed prominent enrichment of the peroxisome proliferator-activated receptor (PPAR) signaling pathway, fatty acid metabolism, and fatty acid degradation, while the bubble plot illustrated their significance levels and gene ratios. The PPAR signaling pathway, enriched with 27 genes, emerged as the most significantly enriched pathway, suggesting that PFOS may interfere with thyroid function by affecting lipid metabolism. In addition, multiple endocrine-related pathways showed significant enrichment, including insulin resistance and adipocytokine signaling pathways, indicating that PFOS may synergistically affect thyroid function by interfering with multiple endocrine pathways. In particular, the significant enrichment of metabolic regulatory pathways, including the AGE-RAGE and AMPK signaling pathways, further supported the hypothesis of PFOS induced thyroid toxicity through disruption of metabolic networks. Moreover, the enrichment of apoptosis and several cancer-related pathways, such as thyroid cancer pathways, suggested that PFOS may cause thyroid toxicity by inducing cell death and carcinogenesis.

### Molecular Docking of PFOS with the core target

This study employed molecular docking analyses to elucidate the interactions between PFOS and five core targets, TP53, JUN, ESR1, AKT1, and CTNNB1. The computational analysis revealed differential binding affinities between PFOS and these target proteins based on AutoDock Vina scoring functions. ESR1 exhibited the most favorable docking score of -9.7 kcal/mol, followed by TP53 with − 8.3 kcal/mol and CTNNB1 with − 7.1 kcal/mol, while AKT1 and JUN showed scores of -6.5 and − 4.9 kcal/mol, respectively. These docking scores suggest potential differences in binding affinity between PFOS and these core targets, with lower scores indicating more favorable predicted interactions.

**Fig. 5:**
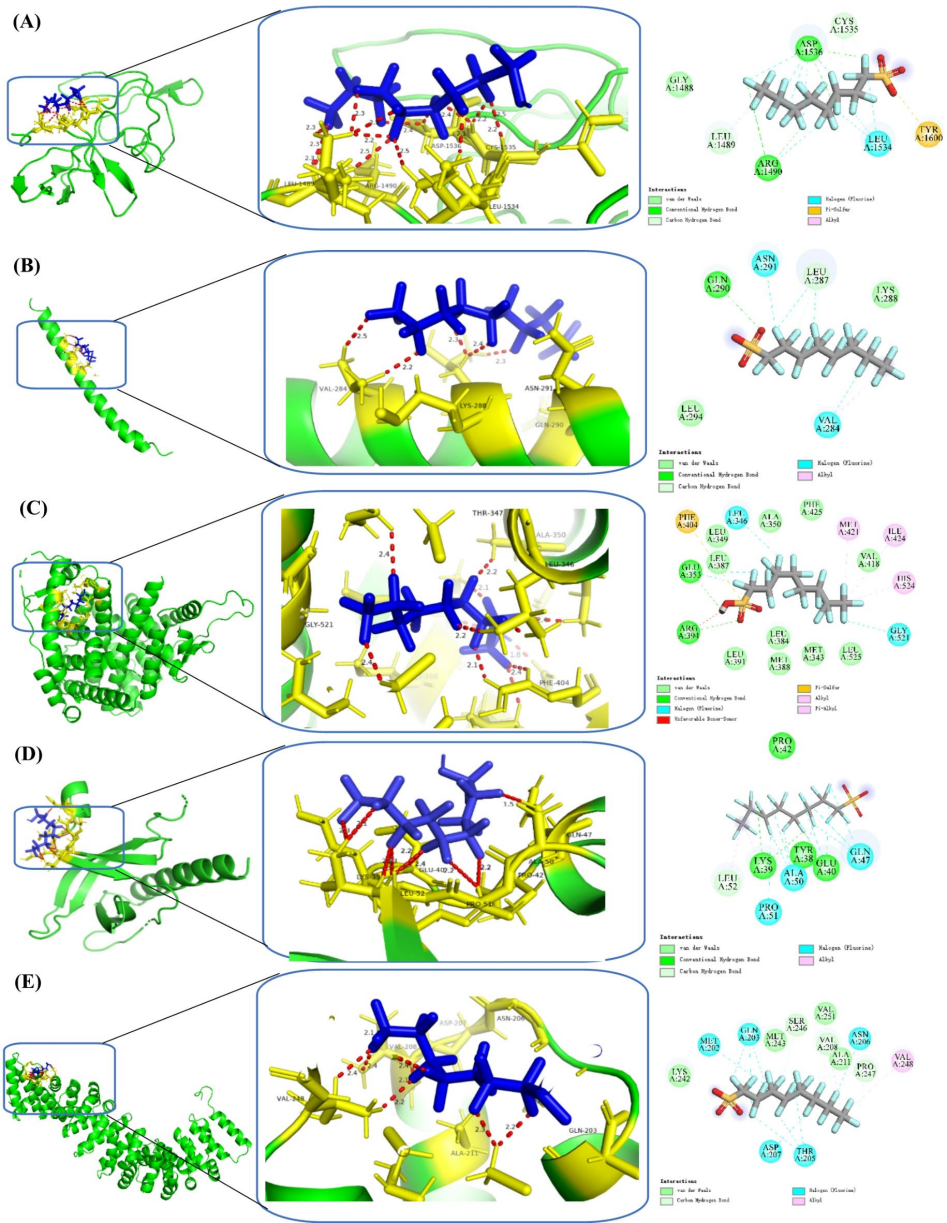
Molecular docking results of the lowest binding energy in each target with PFOS. (**A**) PFOS and TP53; (**B**) PFOS and JUN; (**C**) PFOS and ESR1; (**D**) PFOS and AKT1; (**E**) PFOS and CTNNB1.

Detailed analysis of the molecular docking results revealed distinct binding patterns between PFOS and the target. In the PFOS-TP53 complex (Fig. 5A), interactions are primarily mediated through a combination of van der Waals forces with GLY1488, ALA1459, and CYS1535, alongside hydrogen bonding with ASN1534. The PFOS-JUN complex (Fig. 5B) is stabilized by key interactions with LEU294, VAL284, and ASN291, where the sulfonic acid group forms specific hydrogen bonds with these residues. In the PFOS-ESR1 complex (Fig. 5C), the binding is characterized by an extensive network of interactions, including hydrogen bonds with LEU346, PHE404, and MET343, while the perfluoroalkyl chain establishes hydrophobic contacts within the binding pocket. The PFOS-AKT1 complex (Fig. 5D) shows specific interactions with LYS52, ALA230, and GLU234, where the sulfonic acid group and perfluoroalkyl chain contribute to binding stability. The PFOS-CTNNB1 complex (Fig. 5E) demonstrates interactions primarily through residues ASP202, THR205, and VAL208, forming a combination of hydrogen bonds and hydrophobic contacts. The binding energies and interaction patterns observed suggest potential mechanisms through which PFOS may interfere with the normal functions of these critical regulatory proteins.

### Molecular dynamics simulation of PFOS and core target

**Fig. 6:**
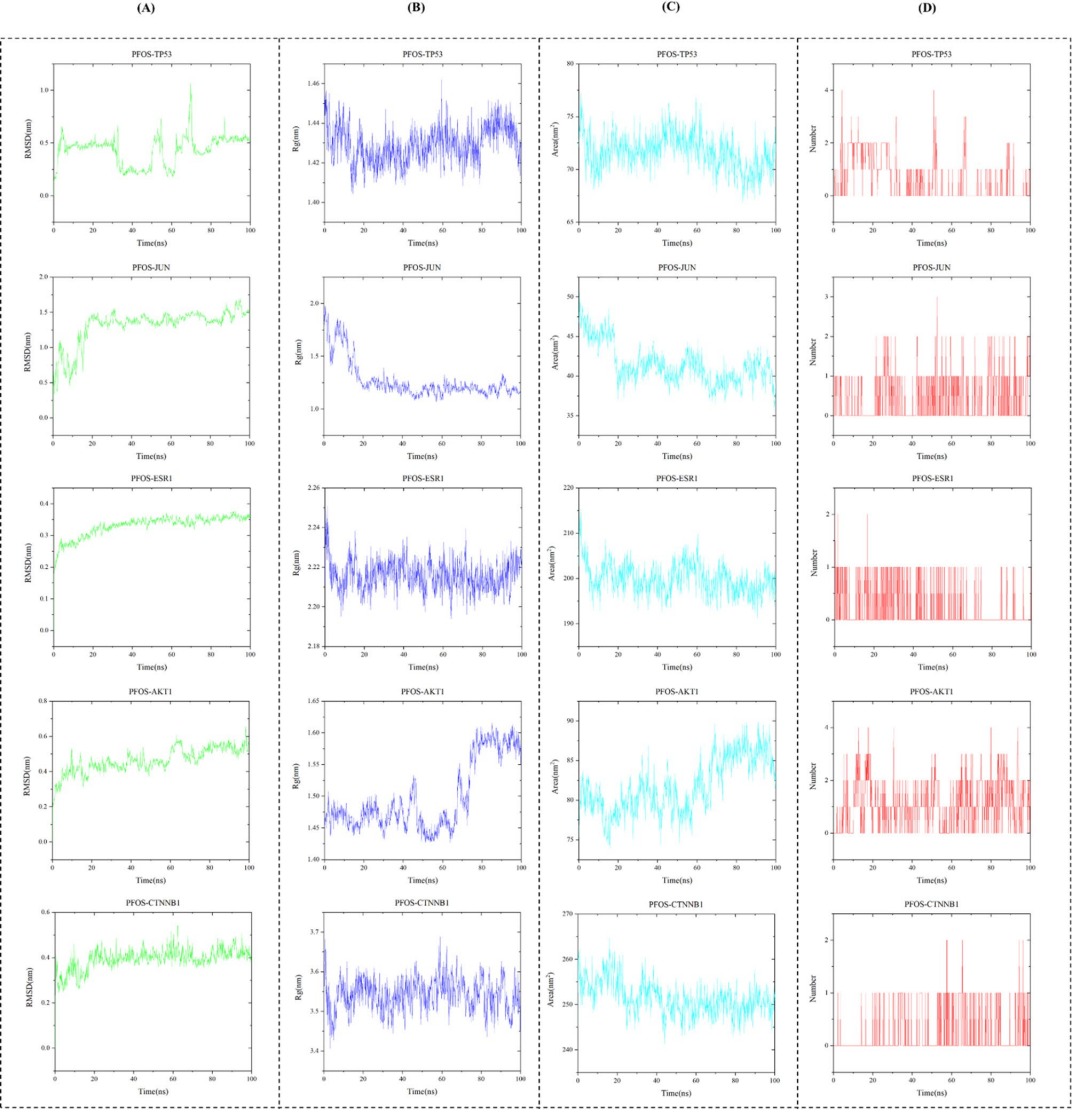
Molecular dynamics simulation results of PFOS with five key target proteins. (**A**) RMSD of backbone atoms during 100 ns simulation for PFOS-TP53, PFOS-JUN, PFOS-ESR1, PFOS-AKT1, and PFOS-CTNNB1 complexes. (**B**) Rg analysis over the simulation time shows the compactness of each complex structure. (**C**) SASA fluctuations during the simulation period indicate the exposure of binding sites. (**D**) Several hydrogen bonds formed between PFOS and target proteins throughout the simulation, demonstrating the stability of protein-ligand interactions. Green lines represent RMSD values, blue lines represent Rg values, cyan lines represent SASA values, and red bars indicate the number of hydrogen bonds. All parameters were monitored over 100 ns molecular dynamics simulation time.

Molecular dynamics simulations of five systems, PFOS-TP53, PFOS-JUN, PFOS-ESR1, PFOS-AKT1, and PFOS-CTNNB1, were performed to evaluate the structural characteristics of PFOS complexes with the core target. As illustrated in Fig. 6A, the RMSD analysis showed that the complexes generally reached conformational equilibrium after initial fluctuations. While most complexes maintained relatively stable RMSD values (PFOS-JUN at 1.41 ± 0.08 nm, PFOS-ESR1 at 0.34 ± 0.01 nm, PFOS-AKT1 at 0.48 ± 0.06 nm, and PFOS-CTNNB1 at 0.41 ± 0.03 nm), the PFOS-TP53 complex showed more dynamic behavior with fluctuations around 0.43 ± 0.15 nm throughout the simulation. Rg analysis demonstrated distinct structural characteristics among the complexes throughout the simulation (Fig. 6B). The PFOS-JUN complex showed the most compact structure with Rg value of 1.19 ± 0.05 nm, while PFOS-CTNNB1 exhibited a more extended conformation with Rg value of 3.54 ± 0.04 nm. PFOS-TP53, PFOS-AKT1, and PFOS-ESR1 displayed intermediate Rg values of 1.42 ± 0.01 nm, 1.51 ± 0.06 nm, and 2.21 ± 0.01 nm, respectively.

SASA analysis was performed to assess the solvent accessibility of the binding interfaces and structural compactness of the complexes (Fig. 6C). Throughout the simulation, all complexes maintained stable SASA profiles, with values of 71.58 ± 1.68 nm², 40.47 ± 1.53 nm², 199.50 ± 3.04 nm², 82.48 ± 3.32 nm², and 250.42 ± 3.08 nm² for PFOS-TP53, PFOS-JUN, PFOS-ESR1, PFOS-AKT1, and PFOS-CTNNB1, respectively. The stability of these SASA values over time suggests that the binding interfaces between PFOS and the target proteins remained consistently organized, indicating the maintenance of stable protein-ligand interactions throughout the simulation period. The hydrogen bond analysis demonstrated distinct interaction patterns for each complex (Fig. 6D). Throughout the 100 ns simulation, each complex showed characteristic hydrogen bonding behavior. The number of hydrogen bonds fluctuated over time, with all complexes maintaining an average of 0–4 hydrogen bonds during the simulation period. These hydrogen bonding patterns, in conjunction with RMSD, Rg, and SASA analyses, elucidate the molecular mechanisms underlying PFOS-protein complex formation and stability.

## Discussion

This study employed integrative analysis to reveal the complex molecular network underlying PFOS induced thyroid toxicity. The study identified 34 core regulatory targets, which demonstrated not only high network centrality but also close functional associations. Identifying these core targets provides novel perspectives for understanding the mechanisms of PFOS-mediated thyroid toxicity. The network topological feature analysis revealed that key regulatory molecules, including TP53, JUN, ESR1, AKT1, and CTNNB1, occupied central positions within the network, suggesting their pivotal roles in PFOS induced thyroid toxicity. Further investigation demonstrated that these core targets primarily involve biological processes, including hormonal response, cellular apoptosis, oxidative stress, and metabolic regulation. These processes correlate strongly with the clinical manifestations of PFOS-induced thyroid toxicity. The pathway analysis revealed significant enrichment in PPAR signaling, lipid metabolism, and endocrine regulatory pathways, indicating that PFOS may induce thyroid dysfunction through interference with an interconnected network of multiple signaling pathways. The findings obtained from the network analysis suggest a complex mechanism whereby PFOS exerts its thyroid toxicity by simultaneous modulation of multiple regulatory nodes rather than through a single molecular target. The system’s understanding of PFOS toxicity mechanisms could have important implications for developing targeted interventions and therapeutic strategies.

The core targets revealed through network analysis occupy crucial positions in topological structure and, more significantly, demonstrate profound biological implications. These core targets are predominantly involved in critical biological processes, including transcriptional regulation, signal transduction, and cell fate determination, suggesting that their functional alterations may directly impact normal thyroid physiology. The core targets identified through network analysis demonstrate remarkable biological significance. TP53 may appear to play a pivotal role in PFOS-induced thyroid toxicity. As a crucial transcription factor, TP53 contributes to thyroid homeostasis by regulating cell cycle progression, DNA damage repair, and apoptotic processes^48^. Molecular docking analyses revealed a substantial binding affinity between PFOS and TP53, with a binding energy of -8.3 kcal/mol. This direct interaction may modulate the transcriptional regulation function of TP53, potentially resulting in abnormal thyroid cell proliferation or apoptosis^49^. This finding is consistent with previous studies reporting that PFOS can induce apoptosis of thyroid cells^50^.

JUN, an AP-1 transcription factor complex component, regulates cellular stress responses, proliferation, and apoptosis^51^. PFOS may directly interact with JUN, potentially modulating its activity and altering the expression profile of downstream genes, particularly those involved in oxidative stress response and apoptotic pathways. This interaction may constitute an essential molecular mechanism through which PFOS induces thyroid toxicity by triggering oxidative stress and inflammatory responses, ultimately promoting thyroid cell damage and apoptosis.

ESR1, a steroid hormone receptor superfamily member, is vital in maintaining endocrine homeostasis, influencing both reproductive system regulation and thyroid hormone synthesis and secretion. PFOS may interact with ESR1 binding sites and disrupt its normal transcriptional regulatory function. This interference may affect the expression or function of thyroid hormone receptors, leading to dysregulation of the thyroid axis^52^. This mechanism elucidates the correlation between PFOS exposure and the alterations in thyroid hormone levels observed in previous studies^53^. In addition, aberrant ESR1 expression may promote thyroid cell proliferation dysregulation, potentially increasing thyroid cancer risk^54^.

AKT1, a key kinase in the PI3K/AKT signaling pathway, is involved in cell survival, proliferation, and metabolic processes. The analysis suggested that PFOS may interfere with thyroid cell energy metabolism and survival signaling through modulation of AKT1 activity^55^. This interaction may contribute to PFOS induced hypothyroidism, as PI3K/AKT pathway dysfunction has been implicated in thyroid hormone synthesis disorders^56^. CTNNB1 is a central effector molecule of the Wnt signaling pathway and plays a key role in thyroid cell proliferation and differentiation^57^. The findings suggest that PFOS may disrupt thyroid follicular cell development and function maintenance through interference with CTNNB1-mediated Wnt pathway activation^58^.

The induction of thyroid toxicity by PFOS appears to be mediated through the modulation of multiple key targets and signaling pathways that form an integrated molecular regulatory network. For instance, TP53 can interact with ESR1 to coordinately regulate the expression of cell cycle-related genes, while AKT1 can regulate JUN activity through phosphorylation modification, thereby regulating cellular stress responses. This complex interaction network indicates that the thyroid toxic effect of PFOS may result from the synergistic action of multiple signaling pathways rather than the alterations in a single path. KEGG enrichment analysis revealed that PFOS induced thyroid toxicity involves the coordinated regulation of multiple critical signaling pathways. Notably, the significant enrichment of the PPAR signaling pathway highlights its important role in PFOS induced thyroid toxicity. PPARs, as essential metabolic regulators, modulate lipid metabolism, energy homeostasis, and inflammatory responses. Hyperactivation of PPAR signaling has been recognized to be closely related to the effects of endocrine disruptors^59^. Previous studies have demonstrated that PFOS can alter the synthesis and metabolism of thyroid hormones by binding to PPAR receptors and modifying fatty acid metabolism^60^. The three major PPAR subtypes are PPARα, PPARβ/δ, and PPARγ, which play distinct roles in these processes. Among them, the activation of PPARα after PFOS exposure is important for the transport, oxidation, and metabolism of fatty acids. Furthermore, aberrant activation of PPAR signaling pathways is closely associated with inflammatory responses, insulin resistance, and metabolic syndrome, which may play pivotal roles in PFOS induced thyroid toxicity^61^.

The significant enrichment of fatty acid metabolism and degradation pathways further supports the hypothesis that PFOS affects thyroid function through metabolic network disruption. Fatty acids, as crucial components of cell membranes, are intricately involved in various physiological processes. After PFOS exposure, fatty acid oxidation and metabolism regulated by PPAR receptors may be altered, leading to fatty acid accumulation and metabolic imbalance^62^. The synthesis, transport, and metabolism of thyroid hormones depend on a typical lipid metabolism environment^63^. Among the potential targets identified in this study, the presence of multiple cytochrome P450 family members (CYP1A1, CYP1A2, CYP2E1, and CYP3A4) suggests that PFOS may disrupt thyroid physiological processes by affecting lipid homeostasis through modulation of these metabolic enzymes. For instance, synthesizing iodothyronine, a thyroid hormone precursor, requires lipid peroxidase participation, while thyroid hormone transport primarily relies on lipoprotein carriers such as transthyretin^64^. Moreover, the accumulation of fatty acids may trigger oxidative stress, further damaging thyroid tissue and cellular function.

The significant enrichment of the Advanced Glycation End-products and their Receptor (AGE-RAGE) signaling pathway indicates its potential involvement in the thyroid toxicity of PFOS. The interaction between AGE-RAGE plays an essential role in metabolic diseases, with pathway activation inducing oxidative stress and inflammatory responses^65^. The significant enrichment of the AMPK signaling pathway reveals the importance of energy metabolism regulation in the toxic effects of PFOS. AMPK, functioning as a cellular energy sensor, is pivotal in regulating lipid metabolism, glucose metabolism, and cellular growth^66^. The findings suggest that PFOS may disrupt the energy homeostasis of thyroid cells by regulating the activity of the AMPK pathway. In particular, the interaction between AMPK and PPAR signaling pathways indicates that PFOS may induce thyroid toxicity through coordinated effects on multiple metabolic regulatory pathways. Further analysis showed extensive interactions between these signaling pathways, forming a complex regulatory network. For example, the PPAR pathway can affect AMPK activity by regulating lipid metabolism, while AMPK activation can reciprocally modulate PPAR transcriptional activity^67^. This complex feedback regulation network suggests that the thyroid toxicity of PFOS may be the result of the synergistic action of multiple mechanisms. Specifically, the combined effects of metabolic dysregulation, oxidative stress, and inflammatory responses may lead to more severe thyroid toxicity^68,69^. It is worth noting that abnormalities in these pathways affect thyroid function directly and impact multiple organ systems through endocrine-metabolic axis interactions. For instance, abnormal lipid metabolism may affect the transport of thyroid hormones, while the activation of inflammatory responses may disrupt the feedback regulation of the pituitary-thyroid axis. This systemic impact potentially explains the complex toxic manifestations caused by PFOS exposure.

Therefore, PFOS induced thyroid toxicity represents a complex process involving multiple targets and pathways acting in concert. This intricate toxicity network encompasses direct cellular damage effects and alterations across numerous dimensions, including metabolic regulation, oxidative stress, and inflammatory responses. This understanding holds significant implications for comprehending the systemic toxic effects of PFOS and developing potential intervention strategies. Furthermore, this synergistic multi-mechanism action suggests that assessing the health risks of environmental pollutants such as PFOS requires a more comprehensive research approach that considers the interactions between multiple toxicity pathways.

Molecular docking analyses produce specific binding patterns between PFOS and core regulatory proteins. From the perspective of structural biology, the binding sites of PFOS are primarily located in the functional key regions of these proteins. The binding site in ESR1 involves key residues near the ligand recognition pocket, potentially directly affecting ESR1’s transcriptional regulatory function. Similarly, the binding of PFOS to TP53 may alter its DNA binding capacity through conformational changes. In addition, although the binding energy of PFOS to JUN is relatively weak at -4.9 kcal/mol, potential physiological effects cannot be excluded when considering the dynamic cellular environment and possible synergistic interactions. This observation underscores the importance of considering multiple factors rather than relying solely on binding energy data when evaluating environmental pollutant toxicity.

The molecular dynamics simulations revealed diverse binding characteristics between PFOS and different target proteins. Analysis of the PFOS-TP53 complex showed dynamic behavior with RMSD fluctuations around 0.43 ± 0.15 nm, suggesting conformational flexibility in the binding interface. The PFOS-ESR1 complex maintained consistent structural parameters (RMSD 0.34 ± 0.01 nm, Rg 2.21 ± 0.01 nm), indicating a stable binding mode that could affect estrogen signaling pathway regulation. The PFOS-AKT1 complex demonstrated distinct structural features (RMSD 0.48 ± 0.06 nm, Rg 1.51 ± 0.06 nm), with patterns of hydrogen bond formation that suggest specific interactions at the binding interface. The PFOS-JUN complex showed unique conformational characteristics (RMSD 1.41 ± 0.08 nm, Rg 1.19 ± 0.05 nm), while the PFOS-CTNNB1 complex exhibited its own distinct binding pattern (RMSD 0.41 ± 0.03 nm, Rg 3.54 ± 0.04 nm).

SASA analysis provided additional insights into the binding interface characteristics of these complexes. The varying SASA values observed across different complexes ranging from 40.47 ± 1.53 nm² to 250.42 ± 3.08 nm² suggest diverse binding pocket architectures and interaction patterns. These molecular dynamic characteristics illuminate the complex nature of PFOS interactions with various protein targets, potentially explaining its broad impact on cellular signaling pathways. This analysis reveals that PFOS can form stable complexes with multiple protein targets through distinct molecular mechanisms, which may contribute to its diverse biological effects. The observed variations in structural parameters and interaction patterns suggest that PFOS’s impact on cellular function may occur through multiple, target-specific mechanisms rather than a single common pathway.

This study integrates network toxicology, molecular docking, and molecular dynamics simulation approaches to construct a research framework for analyzing the mechanism of PFOS-induced thyroid toxicity from the system to the molecular level. The results revealed the multi-level and complexity of PFOS toxicity and emphasized the importance of multi-target and multi-pathway synergy. However, there are still some limitations to this study. Primarily, molecular docking and dynamics simulations mainly focused on the top-ranked targets, which may have overlooked other potentially important interactions. Additionally, the computationally predicted PFOS-protein interaction patterns and their effects on protein function require validation through molecular biology experiments.

## Conclusions

This study employed an integrated computational approach combining network toxicology, molecular docking, and molecular dynamics simulations to elucidate the molecular mechanisms of PFOS induced thyroid toxicity systematically. Potential thyroid toxicity targets were identified through multi-database integration analysis and network topology screening, and core regulatory molecules were determined. The GO and KEGG pathway enrichment analyses demonstrated that the thyroid toxicity of PFOS exhibited multi-target and multi-pathway characteristics. Molecular docking and dynamics simulation studies further confirmed the binding capability and stability of PFOS with these core target proteins, highlighting the importance of hydrogen bonding and hydrophobic interactions in PFOS-protein interactions. These findings provide new insights into the thyroid toxicity mechanism of PFOS and a theoretical foundation for subsequent experimental verification and the development of intervention strategies. Future research should focus on validating the predicted key targets and signaling pathways, exploring network regulation-based protective strategy, and providing scientific evidence for understanding the toxicity mechanism of PFOS and developing protective measures.

## Data Availability

The datasets used and/or analyzed during the current study available from the corresponding author on reasonable request.
